# Planning diabetic retinopathy services — lessons from Latin America

**Published:** 2011-09

**Authors:** Fernando Barráa von-Bischhoffshausen, Francisco Martinez Castro, Pedro Gomez-Bastar

**Affiliations:** Chair, Prevention of Blindness Committee, Pan-American Association of Ophthalmology (PAAO); Chair, Subcommittee on Diabetic Retinopathy, VISION 2020 Latin America; CBM Medical Adviser and Chairman, Instituto de la Vision, Universidad de Montemorelos, Cabrera Num. 206, Colonia del Maestro, Montemorelos, NL Mexico, CP 67510

**Figure:**
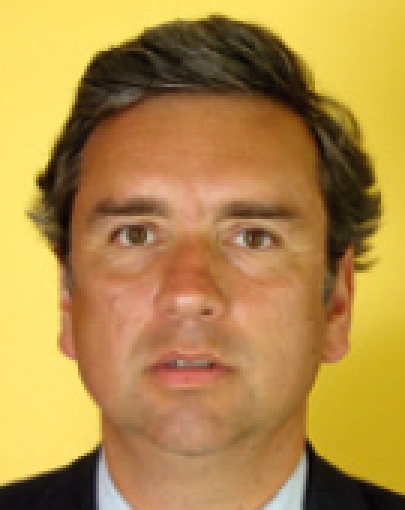


**Figure:**
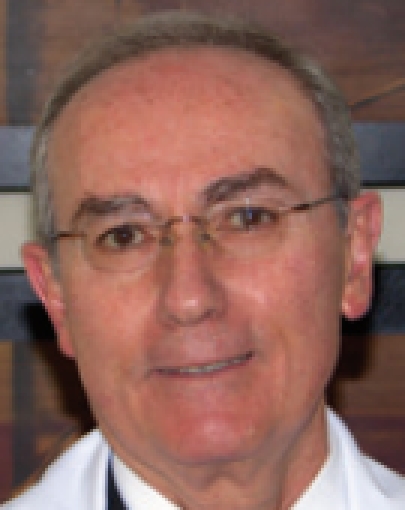


**Figure:**
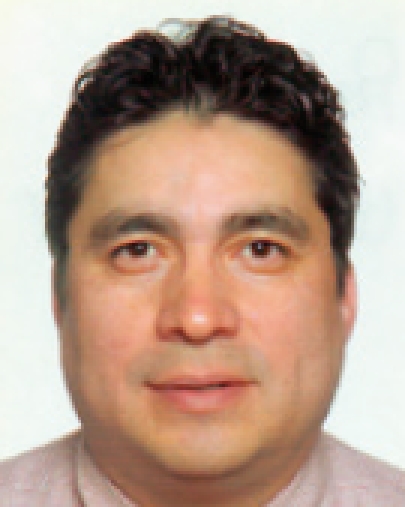

**Key learning points**

- A diabetic retinopathy (DR) programme involves more than finding patients at risk of DR. There must be agreed guidelines on who should be examined, referred, or treated. An accurate register of patients with diabetes is essential, and may be difficult to develop.
- Retinal examination methods should be accurate, cost-effective, and cause minimal inconvenience for the patient. Both retinal photography and retinal examination by an ophthalmologist are accurate, but photography may be more cost-effective in the long-term.
- A referral network is essential so that any patient with diabetes found to have severe retinopathy is guaranteed to receive laser treatment if required.
- Ophthalmologists should work closely with physicians and others to ensure that all patients receive appropriate eye care, and diabetes management, to prevent blindness.

The World Health Organization encourages the promotion and development of programmes for the prevention, detection, and management of diabetic retinopathy (DR). Such programmes must identify effective strategies and technology so that they can be adapted to the situation in each part of the world. Programmes must also be monitored and continuously improved.

The guidelines discussed in this article were developed by experts brought together during workshops hosted by the VISION 2020 Latin America technical subcommittee on DR and technical support was provided by the Pan-American Asociation of Ophthalmology (PAAO). Although these guidelines have been developed for Latin America, we hope that the principles they contain will provide a good starting point for the planning of DR services in other low- and middle-income countries.

## Getting started

Before we start planning a DR programme, it is helpful to review where we are and where we want to be:

- What is the need for DR services (for prevention, diagnosis and treatment) in our population?
- What services and resources are required to meet this need?
- What services and resources are already available, and where do these fall short of the need?

Doing so will allow us to set goals and establish priorities for action.

A programme to manage DR should include the following:

- A good understanding of the current and projected **prevalence of DR**, to make it possible to plan services for prevention, screening, and treatment
- **Clinical guidelines** with a simple classification system, recommended examination intervals, and suggestions for treatment
- A way of **finding patients with diabetes and DR**
- **Retinal examination methods** that take into account available equipment and human resources
- Creation or identification of **laser treatment centres** for timely treatment
- An **education and prevention programme** that reaches the whole population
- **Advocacy** to secure the support of the authorities, educators, general practitioners, endocrinologists, and so on
- **Long-term sustainability**, using cost recovery or subsidies (see article on page 17 for an example from India).

**Figure:**
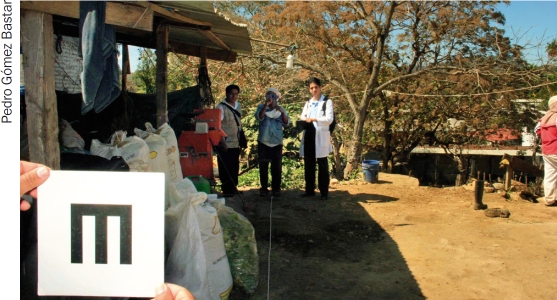
When examining people with diabetes, test their visual acuity before you examine their retinas. MEXICO

## Estimating prevalence

The prevalence of DR can be difficult to estimate, and few estimates have been made in low- and middle-income countries. A survey methodology called RAAB+DR has been developed to estimate the prevalence of DR in a population in a quick and affordable way. RAAB+DR has been tested in Mexico, South Africa, and Saudi Arabia, and the results and recommendations will be discussed in a future issue of this journal.

The prevalence of DR in Latin America was estimated in 1999. At the initiative of the Pan-American Association of Ophthalmology, 7,715 patients with diabetes from 16 countries were assessed. The study found that 40.2% showed some degree of DR, that 17% needed treatment, and that, most worryingly, 35% had never before been examined by an ophthalmologist. A recent population-based study in Mexico found that the prevalence of diabetes in people aged 50 or over was 21%. A total of 39% of patients with diabetes had some DR, 16% had diabetic maculopathy, and 8.6% had proliferative DR. Less than half of those known to have diabetes had been advised to have an annual eye examination.

## Developing clinical guidelines

It is important to have a simple, easy-to-use grading or classification system to help standardise appropriate management, referral, treatment, and monitoring for patients with diabetes. On page 12 of this issue, we have published one such system, based on the international clinical disease severity scale for DR and diabetic macular oedema as set out by the International Council of Ophthalmology (see Useful Resources on page 23).

## Finding patients with diabetes and DR

Ideally, there should be an effective information system that identifies people with diabetes, calls them for screening, and records the outcomes of eye examinations and/or referrals. In Latin America, because of its many fragmented health care systems, identifying patients with diabetes for a national or regional screening programme poses a difficult challenge.

**Figure:**
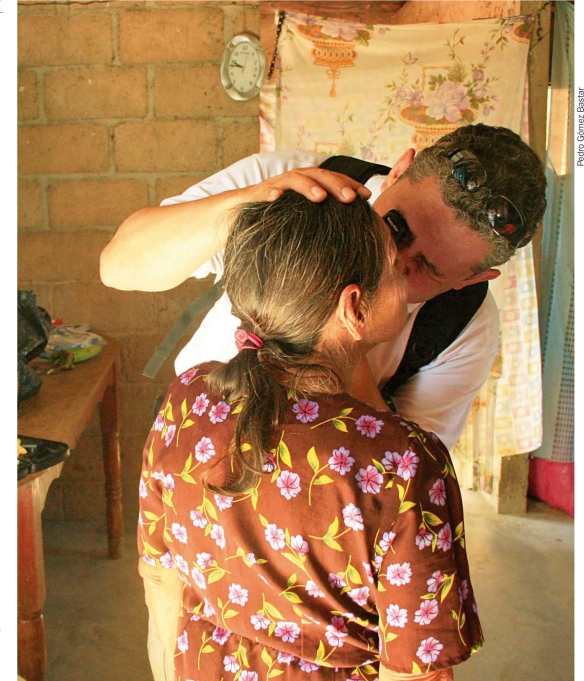
Examining a patient's retina. MEXICO

Any screening programme requires clear referral criteria; only patients with retinopathy meeting a pre-defined threshold should be referred to ophthalmologists. In addition, there has to be some quality control to ensure that the screening programme is effective. In areas where services are available, all patients diagnosed as having diabetes should be examined. If that is not possible, we should consider concentrating on high-risk groups, with priority given to people with type 1 diabetes, people aged 50 or over, those with type 2 diabetes of more than ten years' duration, pregnant women with gestational diabetes, and patients with nephropathy (which can be detected by testing for the presence of albumen in the urine).

## Retinal examinations

Yearly retinal examinations of all diabetes patients are necessary because the condition is asymptomatic in its early stages, and because early treatment reduces both the risk of blindness and the cost of treatment. Methods of detection include the following:

1. **Retinal examination with a slit lamp and hand-held lens following pupil dilation**. This is the method with the greatest specificity (it does not tend to wrongly classify someone who in fact does not have DR as having DR) and sensitivity (it does not tend to miss DR in someone who in fact has it). However, it is time-consuming and hence costly.
2. **Taking one or two photographs of each eye with a non-mydriatic camera**. This achieves good sensitivity and specificity. Retinal photography with a digital fundus camera is rapid and sensitive. Although the camera is expensive, it may reduce costs as only patients with positive findings are referred to ophthalmologists. The photographs can betaken by technicians, allowing ophthalmologists to examine the photographs of large numbers of patients in a short time.
3. **Using a direct or indirect ophthalmoscope**. This has less sensitivity but is useful when you do not have a slit lamp and lens.

**‘Retinal examination methods should be accurate, cost-effective, and cause minimal inconvenience for the patient’**

## Treatment

DR requires **early treatment** to slow or stop the progression of the disease. Improved control of diabetes (page 4) is the most important, especially in patients with diabetic macular oedema. Patients who have established sight-threatening retinopathy will require laser treatment. Steroids and intravitreal anti-vasculuar endothelial growth factor (anti-VEGF) therapy are used together with laser therapy for macular oedema. Vitrectomy is indicated for non-clearing vitreous haemorrhage and tractional retinal detachment.

A workable DR programme must have the facilities, equipment, consumables, medicines, and staff to provide all of the above.

## Education and prevention

Education is a priority for the prevention of blindness due to diabetic retinopathy. There must be clear messages for people with diabetes, their families, health workers, and the general public, along the following lines:

- DR is asymptomatic (it has no symptoms), and it carries a real risk of blindness.
- With annual examination of the retina, early detection, and prompt laser treatment, sight can normally be preserved.
- Strict control of diabetes and blood pressure reduces the risk of retinopathy.

At the primary care level, education should focus on lifestyle and prevention of diabetes by diet and exercise. At the secondary level, education should encourage better self-care by patients, including improved control of blood sugar and blood pressure (see article on page 4). Education should also promote regular eye examinations for all people with diabetes.

## Planning and advocacy

Effective lobbying or advocacy is essential. Advocacy is the act of arguing on behalf of a particular cause, such as establishing a new DR programme, with the aim to influence decision makers to support this cause. When planning an eye care programme, you should aim to develop a solution that is appropriate to the local situation and that is directed at the population with the greatest needs. Aim to ensure the greatest possible coverage, quality of care, and sustainability in the long term.

Political will is needed in order to implement eye health policies, and this can be generated by effective advocacy. Ideally, the eye care programme should be developed by a working group in which everyone involved in the project is represented. This group can identify any decision makers whose support will be required and invite them to participate. The earlier the decision makers are involved in designing the solution, the more likely they are to support the outcome and make helpful contributions. This turns an obstacle (“How will we get their support?”) into an opportunity for collaboration.

**‘Political will is needed in order to implement eye health policies, and this can be generated by effective advocacy’**

Any current inability to meet the existing demand for ophthalmological services is fertile ground for promoting our DR programmes. In Latin America, we can deliver clear messages to the health care authorities or legislators along the following lines:

1. Diabetes affects 7-10% of the population over the age of 20. Through screening, we may find retinopathy in as much as 30% of patients with diabetes, and 5% of patients with diabetes are likely to need laser treatment to reduce the risk of blindness.
2. Diabetes will be increasing in the future, and it is around twenty times cheaper to treat it earlier rather than later.
3. Eye health plans should be directed toward helping the most vulnerable people so as to achieve equal access to health care.

It is important to describe and publish the results of current and past prevention of blindness programmes. Publishing in scientific journals helps to provide the evidence you may need to convince decision makers. Persuading the media (newspapers, radio and television) to then write and talk about this evidence creates public pressure that will also persuade decision makers to act.

In Latin America, the epidemic of diabetes and DR poses such a great challenge to the population's health that we cannot manage alone. Through the leadership of the ophthalmology societies of Latin America, supranational bodies such as the Pan-American Association of Ophthalmology (PAAO), and other organisations such as the Pan-American Health Organization (PAHO) and the International Agency for the Prevention of Blindness (IAPB), we can forge alliances with national governments. These alliances, when added to the initiatives of non-governmental organisations, the ophthalmic industry, and civil society, can greatly assist with the implementation of national plans for the detection and control of DR.

Worldwide, any successful strategy to address DR will require close collaboration among everyone concerned: ophthalmologists, endocrinologists, physicians, mid-level eye care workers, outreach workers, pharmacists, public health specialists, community leaders, politicians, diabetes patients, and the general public.

There is a lot to do, but together we can do it!

**Finding diabetes patients: thinking beyond the eye clinic**

We can do nothing about diabetes or diabetic retinopathy (DR) unless we know where to find people who have diabetes.

Screening programmes are expensive, and countries with limited resources should not attempt a national screening programme for DR; it would be too complex and expensive to set up, administer, and manage.

It may be more cost-effective to work closely with our colleagues who see diabetes patients during the course of their work, such as physicians, diabetologists, pharmacists, and health insurers. We must encourage them to look for eye disease in their patients, or at the very least to refer their patients for regular retinal examination (provided that local treatment services are available).

We should also look for diabetes patients in our eye clinics, particularly those patients with cataract, as cataract can be a consequence of diabetes. We must check patients' blood sugar (if possible), carefully examine their eyes, and refer them for follow-up and/or further investigation (see the table on page 12). We must also ensure that they have access to a service to help them manage their diabetes.

However big or small our screening programmes, it is important to focus on more than the clinical and technical aspects (such as camera vs. ophthalmoscope or technicians vs. ophthalmologists). The biggest problems are administrative and managerial:

- How do we identify the diabetes patients we want to examine?
- How do we contact them to come for an examination?
- What do we do if they don't turn up?
- What do we do if a clear enough view of the retina is not possible?
- How do we record the findings, and how do we share that information? With whom do we share it, and when?
- Where and how are patients referred?
- How many of the people needing treatment actually attend and accept treatment after referral?
- What is the outcome of treatment?

In order for our screening to succeed, it is important to address these questions as early as possible in any planning process.

**Experiences in India**

Dr Rajiv Raman and his colleagues in India have reported that only 54% of the general practitioners or physicians they studied were aware of the need for annual retinal examinations and referral for patients with diabetes. Just 1.3% used direct ophthalmoscopes to detect DR, of which only half dilated patients' pupils before examination. The barriers they faced were lack of time, lack of ophthalmoscopes and lack of training.

According to Dr Raman, diabetes patients in India also regularly visit their pharmacists. Dr Raman recommends creating awareness among general practitioners and pharmacists about their role in identifying and referring patients at risk of DR. General practitioners could also be trained in the use of a direct ophthalmoscope as part of their continued medical education or continued professional development.

